# Adsorption of Cationic Lignin Derivatives on Negatively Charged Model Surfaces and Hair Fibers: Implications for Hair Conditioning Performance

**DOI:** 10.1021/acsami.6c09520

**Published:** 2026-06-29

**Authors:** Catarina Fernandes, Daniela Cabaça, Eduardo Guzmán, Ricardo Serra, Alireza Eivazi, Magnus Norgren, Luís Alves, Bruno Medronho, Maria da Graça Rasteiro, Carla Varela

**Affiliations:** † University of Coimbra, CERES, Department of Chemical Engineering, Pólo II − R. Silvio Lima, 3030-790 Coimbra, Portugal; ‡ MED Mediterranean Institute for Agriculture, Environment and Development &CHANGE Global Change and Sustainability Institute, Universidade do Algarve, Faculdade de Ciências e Tecnologia, Campus de Gambelas, Ed.8, 8005-139 Faro, Portugal; § Complutense University of Madrid, Department of Physico Chemistry-Plaza de Las Ciencias s/n, 28040 Madrid, Spain; ∥ Complutense University of Madrid, Pludisciplinar Institute-Paseo Juan XXIII 1, 28040 Madrid, Spain; ⊥ CEMMPRE Mechanical Engineering Department, 37829 University of Coimbra, 3030-788 Coimbra, Portugal; # Surface and Colloid Engineering, FSCN Research Center, Mid Sweden University, SE-851 70 Sundsvall, Sweden

**Keywords:** biobased polymers, lignin valorization, cosmetic ingredients, hair care, polymer adsorption, polyquaternium, surface charge

## Abstract

The application of cosmetic ingredients into hair formulations relies on their extensive characterization and on understanding their mechanisms of action. Specifically, in the case of hair conditioning agents, their efficiency in treating hair must be proved before testing them on real complex formulations. In this work, we investigate the deposition of three cationic polymers onto model surfaces that mimic the negative surface potential of highly damaged hair. Two CHPTAC-cationized lignins (CL0.34 and CL0.61) were evaluated and compared with a commercial polyquaternium (PQ11). The two selected lignin derivatives exhibited different degrees of cationic substitution (DS) and ζ-potential (CL0.34: DS = 0.34 ± 0.01 and ζ-potential = 12.8 ± 0.4 mV; CL0.61: DS = 0.61 ± 0.03 and ζ-potential = 18.8 ± 0.3 mV). Atomic force microscopy (AFM) and quartz crystal microbalance with dissipation monitoring (QCM-D) were used to evaluate the adsorbed layers formed by the polymers and their mechanical properties. Among the tested lignin conditioning agents, CL0.61 exhibited conditioning behavior, forming layers whose properties closely resembled those of the benchmark polymer PQ11. CL0.61 and PQ11 were both efficient at reducing the frizz effect on real bleached hair, effectively overcompensating the hair surface potential, which shifted from negative to positive values, confirming their effective adsorption after conditioning and rinsing. By combining advanced interfacial characterization with structure–property–function relationships, this work provides fundamental insights into polymer adsorption and performance at biointerfaces, supporting the rational design of functional materials and highlighting the potential of cationic lignin derivatives as viable, biobased conditioning agents for future hair-care formulations.

## Graphical abstract

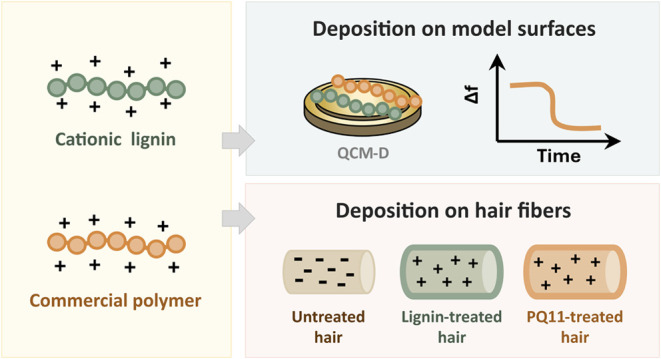

## 1 Introduction

Hair is a fibrous material and a component of the integumentary system, responsible for offering thermoprotection and acting as a barrier to UV radiation. Its features, such as color, shape, and luster, contribute to its overall aesthetics,^1^ and, therefore, its emotional and social roles should not be underrated. Hair has a cylindrical, hierarchical structure, and the hair shaft is mainly composed of proteins and lipids.^2^ Depending on its moisture content (which can represent up to 32% of the hair weight), the protein content varies from 65% to 95%,^3^ corresponding to more than 90% of the hair dry weight.^2^ From those proteins, the fibrous and resistant α-helical keratin is of special relevance as it is the main component of the hair cortex. The cortex is enclosed by the cuticle, a structure of flat overlapping scales of proteins and lipids.^4^ Other compounds are also found, including pigments and other minor compounds.^3^

As the outer layer of the hair, the cuticle is highly exposed and affected by external degradation, being the substrate in which hair care products act and remain adsorbed. Cuticle integrity is essential for the aesthetic attributes of hair. The cuticle scales are covered by a lipidic layer mainly composed of 18-methyleicosanoic acid (18-MEA), chemically linked to the protein matrix, which is responsible for the hydrophobic surface of healthy hair and contributes for the combing properties as it acts as a lubricant to reduce friction between hair fibers.^5^ The integrity of this layer is of paramount importance for the physicochemical action of cosmetics since hydrophobic substances, like silicones, fatty alcohols, and polymers, can only favorably interact with the cuticle surface if its hydrophobicity is kept intact.^6^ However, the constant exposure to different stimuli, such as mechanical, thermal, or chemical processes, leads to the depletion of the fatty acids, and to the oxidation of the disulfide bonds from the cystine residues to cysteic acid.^7,8^ These degradation pathways increase the density of negative charges on the surface of damaged hair, enhancing its hydrophilicity. Consequently, the mechanical, visual, and sensory properties of hair get affected, becoming frizzy, dry, and prone to tangling.

Although there are different mechanisms to impart hair conditioning, the treatment of highly damaged hair is majorly accomplished by the deposition of positively charged compounds, such as cationic surfactants or polymers. The physicochemical aspects of the hair conditioning process were recently reviewed.^7^ Briefly, they mainly act through electrostatic attraction between the positively charged groups of cationic surfactants or polymers and the negatively charged surface of highly damaged hair fibers. This results in the formation of conditioning deposits on the hair shaft that contribute to neutralizing the negative charges, decreasing the frizzing effect, and conferring luster and smoothness to hair. Therefore, the ability of a cationic polymer to interact and remain adsorbed onto negatively charged surfaces provides a preliminary assessment of its potential efficiency as a conditioning ingredient.

Given the complexity and heterogeneity of the hair surface across individuals, hair type, and even the region within a single hair fiber, models that mimic its general features, such as surface charge and wettability, have been proposed to perform initial testing.^9,10^ This strategy provides a simplified approach that results in higher reproducibility and easier understanding of the fundamental physicochemical processes that govern the potential performance of polymers in hair conditioning. For instance, damaged hair is characterized by a low amount of bounded lipids and a high amount of oxidized amino acids, which creates a highly negative and hydrophilic surface.^8,11^ Therefore, negatively charged surfaces such as gold, silica, or mica, are frequently used as models for the adsorption studies.^8^ Although negatively charged model surfaces offer a useful and reproducible first approximation of the negatively charged hair surface, more sophisticated model interfaces, such as self-assembled monolayers (SAMs)^12^ and polymer brushes,^13,14^ allow a finer control of the surface chemistry, charge density, and molecular architecture. These platforms can enable more detailed investigation of polymer adsorption and interfacial interactions and may be valuable for future studies aimed at approaching the complexity of real biological surfaces. Techniques such as atomic force microscopy (AFM) and quatrz crystal microbalance with dissipation monitoring (QCM-D) give access to complementary information related to the adsorbed conditioning deposits, namely the layer topography from AFM, and the adsorbed wet mass and mechanical properties from QCM-D.^15^

From the cationic polymers used in conditioner formulations, the polyquaternium (PQ) family constitutes one of the most commonly employed classes. These cationic conditioning agents have cationic quaternary ammonium groups that allow them to interact with the negative charges of damaged hair and neutralize them.^7,15^ However, these compounds are reported to cause skin and eye irritation and to be highly toxic to aquatic organisms.^16^ In addition, they are usually nonbiodegradable and nonrenewable. These environmental and safety concerns have driven the cosmetic industry to seek alternative conditioning agents derived from renewable resources, capable of maintaining performance while reducing ecological and toxicological impacts.^7^

In this regard, biobased polymers and biopolymers are in the spotlight of the research community as renewable alternatives to petrochemical-based polymers for various applications, including as new cationic conditioning agents. This has fueled the research on biomass-derived polymers, such as lignin, for the hair care sector. These polymers are beneficial due to their biocompatible, ecofriendly, and highly marketable character.^17^ Examples of biobased and biopolymers used in cosmetics include xanthan and guar gums, starch, alginate, collagen, and hyaluronic acid, which are primarily used as modifiers and stabilizers.^17,18^ In particular, chitosan,^19^ cationic amino acid–based surfactants,^20^ and esterquats^7,21^ have been reported to successfully act as conditioning agents due to their cationic nature. In this work, we explore cationic lignin as a new biobased and eco-friendly alternative to conventional hair conditioning agents.

Lignin has an estimated availability in the biosphere of about 300 billion tones,^22^ being the primary aromatic platform relevant for both chemical production and materials development.^23^ Although most lignin is still being used for heat generation, as is the case for the majority of the lignin annually produced by pulp and paper industries,^24^ its use for the development of high-value products has expanded.^25–28^ This includes its increasing application as a cosmetic ingredient due to its appealing characteristics, namely its antioxidant^29,30^ and antibacterial activities,^31,32^ and its ultraviolet radiation filtering capacity.^29,33^ Its cosmetic potential has also driven its application in hair care formulations, as recently reviewed.^7^ For instance, nanolignin, combined with chitin nanofibrils, has been proposed as a carrier for active ingredients in hair repair.^34^ More recently, lignin-based emulsions showed effective lubrication of damaged hair fibers and a reduction in combing force.^35^ However, in these studies, lignin was used as a carrier or an emulsion stabilizer rather than as the active conditioning agent itself. For lignin to be used as a hair conditioning agent, some structural modifications are required, mainly to improve its water solubility and surface charge. In this regard, cationic lignin derivatives can be obtained by grafting quaternary ammonium groups onto the lignin backbone,^36–39^ enabling electrostatic interactions with damaged hair.

Given the abundance, renewability, and nontoxicity of lignin, this work aims to study the use of cationic lignin derivatives as alternatives to petrol-based conditioning agents in hair care products. These cationic derivatives were prepared by modifying acacia lignin^40^ via 3-chloro-2-hydroxypropyltrimethylammonium chloride (CHPTAC) single-step modification process, as described in our previous work.^39^ Two cationic lignin derivatives, CL0.34 and CL0.61, with different physicochemical properties, were selected for further characterization, performing deposition studies using model substrates intended to mimic hair surface. Polyquaternium-11 (PQ11), a copolymer of vinylpyrrolidone containing cationic groups derived from dimethylaminoethyl methacrylate quaternized with diethyl sulfate,^41^ was selected as the petro-based reference commonly used in hair conditioners.

## 2 Materials and Methods

### 2.1 Materials

Lactic acid (88–92%) from Panreac and citric acid (99.9%) from José Manuel Gomes dos Santos, Lda., and choline chloride (>98.0%) from TCI were used for lignin extraction. Cationic lignin derivatives, CL0.34 and CL0.61, were synthesized from acacia lignin as described in section 2.2 using 3-chloro-2-hydroxypropyltrimethylammonium chloride (CHPTAC, 60 wt % aqueous solution) from Sigma-Aldrich (Darmstadt, Germany). Single-side polished, thermally oxidized silicon dioxide wafers (PRO2080 Si 6in n/P SSP 1–30 Res 90 nm SiO_2_) were purchased from PhotonExport (Barcelona, Spain). Sulfuric acid (H_2_SO_4_, 96%), sodium hydroxide (NaOH, 99%), and hydrogen peroxide (H_2_O_2_, 30 wt %) were obtained from José Manuel Gomes dos Santos, Lda. (Odivelas, Portugal) and hydrochloric acid (HCl, 37 wt %) from Fisher Scientific (Porto Salvo, Portugal). Polyquaternium-11 (PQ11, aqueous solution, 22 wt %) was obtained from Derypol (Barcelona, Spain).

### 2.2 Synthesis of Cationic Lignin Derivatives

Cationic lignin derivatives were prepared from lignin extracted from acacia wood (*Acacia dealbata* Link) collected in Coimbra, Portugal. Acacia sawdust was pretreated using the preoptimized extraction method as described in our previous work.^40^ Briefly, lignin was extracted at a solid–liquid ratio of 1:10 (w/w) with a natural deep eutectic solvent (NADES) composed of lactic acid, citric acid, and choline chloride (molar ratio of 0.6:0.3:0.1) for 2 h at 140 °C in a Teflon-lined stainless-steel reactor. The extracted lignin showed a purity of 91.45%.^40^

The lignin cationization was then performed as described in our previous publication.^39^ Briefly, ca. 500 mg of acacia lignin and 1 M aqueous NaOH solution were placed in a round-bottom flask and stirred until lignin was completely solubilized. The quantity of NaOH was adjusted according to the ratios presented in Table 1. After complete dissolution, the selected amount of CHPTAC was slowly added to the lignin-containing solution and let to react for 3 h at the desired temperature. The reaction conditions for each sample are summarized in Table 1. At the end of the reaction, the cationic lignin derivatives were separated from the unreacted CHPTAC by dialysis in water using dialysis membranes with a cutoff of 2 kDa (Spectra/Por prewetted standard RC from Spectrum) after proper neutralization of the alkaline solution with 1 M HCl. The samples were then oven-dried and characterized regarding their degree of substitution (DS), ζ-potential, and viscosity-average molecular weight (Mv).

**Table 1:** Reaction Conditions for the Synthesis of the Cationic Lignin Derivatives and Resulting Physicochemical Characterization^a^,^b^,^c^

	CL0.34	CL0.61
CHPTAC ratio	2.55	1.3
NaOH ratio	1	1.25
temperature (°C)	50	70
degree of substitution	0.34 ± 0.01^b^	0.61 ± 0.03^a^
ζ-potential (mV)	12.8 ± 0.4^b^	18.8 ± 0.3^a^
Mv (kDa)	8.65 ± 1.09^a^	12.79 ± 2.73^a^

a Reaction time was 3 h for both samples. Values sharing the same letter within a row indicate no statistically significant differences between the samples (Tukey test, α = 0.05). Synthesis and characterization data were obtained from our previous optimization study.^39^

b Characterization. CHPTAC ratio = n_CHPTAC_:n_lignin_

c NaOH ratio = n_NaOH_:(n_CHPTAC_ + n_lignin_)

#### 2.2.1 Characterization of CL0.34 and CL0.61

The characterization of the cationic lignins involved the determination of their DS, ζ-potential, and Mv. The DS gives direct information on the efficiency of the cationization by representing the amount of cationic moieties introduced, whereas the ζ-potential is related to the surface charge,^42^ being considered an indicator of the polymer’s effective charge.^43^ For detailed information and discussion on the characterization of cationic lignins, the reader is referred to our previous work regarding the chemical modification of lignin.^39^

The DS of the synthesized lignins were estimated by elemental analysis by determining the nitrogen (N) content. Detailed information on the calculation of the DS of cationic lignins can be found in our previous publication.^39^ The N content was determined using an Organic Elemental Analyzer from NC Technologies (model ECS 8040 CHNS-O) and the DS calculated based on eq (1), where 207.86 g·mol^–1^ is the molecular mass of the lignin monomer,^39^ 14 g·mol^–1^ is the atomic mass of nitrogen and 151.66 g·mol^–1^ is the molecular weight of the introduced cationic group.

1 $$\mathrm{DS}=\frac{207.86\left(g\cdot \mathrm{mo}l^{-1}\right)\times N\left(\%\right)}{14\left(g\cdot \mathrm{mo}l^{-1}\right)\times 100\left(\%\right)-151.66\left(g\cdot \mathrm{mo}l^{-1}\right)\times N\left(\%\right)}$$

The ζ-potential of the samples was determined from electrophoretic mobility data obtained by laser Doppler electrophoresis using a ZN 3500 Zetasizer NanoZS instrument from Malvern Instruments Ltd. (Malvern, U.K.). Ca. 2–3 mg of cationic lignin were dissolved or dispersed in 10 mL of deionized water and then analyzed in duplicate, using a folded capillary zeta cell. A total of six measurements per replicate were performed. The ζ-potential was also determined for the commercial polymer PQ11.

The Mv of the cationic lignins was estimated by the intrinsic viscosity method using an automatic capillary viscometer Viscologic TI1 from Sematech and applying the Mark–Houwink–Sakurada equation.^44^ The detailed procedure and determination of the intrinsic viscosities can be found in our previous work.^39^ Briefly, the flow times of successive dilutions (40 to 10 mg·mL^–1^) of lignin in 0.5 M NaOH were measured at 30 °C. The intrinsic viscosities were obtained by extrapolation to zero concentration using the Huggins relationship.^45^

### 2.3 Studies of Polymer-Hair Interactions Using Model Surfaces

The potential of the synthesized cationic lignin derivatives to act as hair conditioning agents was evaluated by studying their deposition and interaction with model surfaces that are representative of the hair surface. For this, silicon dioxide (SiO_2_) was selected as it has a negatively charged surface that mimics the negative net charge of highly damaged hair.^46^ In this sense, the SiO_2_ substrate should be viewed as a simplified screening interface, while more architecturally complex surfaces, such as SAMs and polymer brushes, could be used in future work to analyze the role of surface charge, hydration, and molecular organization in greater detail.

Prior to use, the silicon wafers were cut to the required size for the analysis (squares of ca. 1 × 1 cm^2^) and thoroughly cleaned using a piranha solution, prepared immediately before use by carefully mixing 96% H_2_SO_4_ and 30% H_2_O_2_ at a 3:1 v/v ratio. The wafers were then soaked in the piranha solution for 30 min and subsequently exhaustively rinsed with deionized water.^47,48^ This cleaning procedure is essential to ensure complete removal of organic contaminants and to guarantee a reproducible, highly hydrophilic surface reminiscent of the surface of highly damaged hair fibers. The cleaned surfaces were used as substrates for the deposition of the conditioning polymers and subsequent topographical studies by AFM. An identical cleaning and preparation protocol was applied to the SiO_2_-coated quartz sensors used in the QCM-D experiments.

#### 2.3.1 Atomic Force Microscopy (AFM)

The topographical images of silicon wafers coated with CL0.34, CL0.61, and PQ11 were obtained by AFM. Initially, aqueous solutions of CL0.34, CL0.61, and PQ11 with a concentration of 0.5 wt % were prepared by dissolving the polymers in 10 mL of deionized water and then poured into a standard 25 mL glass beaker. The cleaned silicon wafers were immersed in these solutions for 5 min and then withdrawn and immersed in a beaker containing 10 mL of deionized water for 1 min. Finally, the wafers with the adsorbed polymers were oven-dried at 40 °C and kept in a desiccator with silica gel until further use.^49^

AFM analysis of the dry polymers deposited onto the silicon surfaces were carried out at room temperature in an AFM Di-innova microscope (Veeco Instrument Inc.) operated in tapping mode. The cantilever used was a silicon n-type (AppNano) with 150 μm length × 28 μm width × 3 μm thickness, at a resonance frequency of 150 kHz and a specified normal spring constant (K) of 7.8 N·m^–1^, with a tip of <10 nm radius and 14–16 μm of height.

#### 2.3.2 Quartz Crystal Microbalance with Dissipation Monitoring (QCM-D)

A QCM-D model Explorer from Qsense (Biolin Scientific, Gothenburg, Sweden) fitted with a quartz sensor coated with a SiO_2_ layer was used to further characterize the adsorption of the cationic polymers on negatively charge surfaces. Milli-Q grade ultrapure deionized water, characterized by a resistivity higher than 18 MΩ·cm and a total organic content lower than 6 ppm, was used for the experiments and for cleaning the materials used. Details on the QCM-D experiments can be found in the work by Fernández-Peña et al.^50^

The adsorption experiments of CL0.34, CL0.61, and PQ11 were performed in a flow cell, consisting of three successive steps. First, the measurement chamber was conditioned by flushing with ultrapure water until a stable baseline was obtained for the frequency and dissipation signals of the quartz resonator. This process was performed for a minimum period of 5 min. Second, the polymer solution (0.5 wt %) was introduced in the chamber, and its adsorption was monitored until both the frequency and dissipation signals reached a new steady state, indicating the end of the adsorption process. Finally, the measurement chamber was flushed with water and maintained at the same temperature of the measurements (25 °C), in order to remove any material weakly adsorbed to the substrate.

### 2.4 Studies of Polymer-Hair Interactions Using Real Hair Samples

To evaluate the potential of the prepared derivatives to interact with human hair and confer the desired conditioning effect, the most promising lignin derivative (CL0.61) and the commercial polymer (PQ11) were also tested in real hair samples. These consisted of chemically damaged hair, previously bleached in a hair salon before the collection. Several identical tresses were prepared by combining and gluing together similar amounts of hair fibers. Five independent tresses were used: one as reference, without any conditioning treatment applied, and four treated with aqueous solutions of the cationic conditioning agents CL0.61 or PQ11. Hair was treated as follows: all five hair samples were thoroughly washed using warm tap water and a liquid soap that did not contain any cationic polymer/surfactant in its composition. After rinsing with more warm tap water, the hair samples were rinsed with ultrapure water and air-dried (reference) or immersed in an aqueous solution containing the conditioning agent of interest (i.e., PQ11 or CL0.61, 1 wt %) for 10 min. At the end, a rinsing step was performed in some of the samples to mimic the real-life scenario of hair rinsing by immersing the treated tresses in 10 mL of water for 2 min. Hair samples were air-dried before further analysis. In summary, two samples were treated with CL0.61 solution, from which, one was then been rinsed with water and the other was allowed to dry immediately after the conditioning step. The same approach was followed for hair samples treated with PQ11. The treatment sequence for all samples is listed in Table 2.

**Table 2:** Treatment Sequence Applied to Hair Samples

sample	1. washing	2. conditioning	3. rinsing
reference	√	—	—
PQ11 not rinsed	√	√	—
CL0.61 not rinsed	√	√	—
PQ11 rinsed	√	√	√
CL0.61 rinsed	√	√	√

#### 2.4.1 Hair Surface Analysis (AFM/KPFM)

Surface morphology and roughness of the hair samples were accessed by AFM (Park Systems NX20, Korea). AFM was operated in noncontact mode in air. A PPP-EFM probe (Park Systems, Korea) with a nominal resonance frequency of 75 kHz and force constant 2.8 N·m^–1^ was used. AFM images of representative areas (10 × 10 μm^2^) on the hair samples were acquired using a scan rate of 0.8–1 Hz, ensuring more than 95% match between forward and backward scans. The surface roughness parameter (R_q_) was determined using the Park Systems XEI 1.8.5 image analysis software. Before analysis, the hair samples were conditioned in an oven at 40 °C for 24 h, and then carefully attached to the metal sample disk using double-sided carbon tape to make electrical contact between the metal sample disk and the sample top surface. The surface potential was obtained using amplitude-modulated Kelvin probe force microscope (KPFM) mode. Before each main measurement, the work function of the tip was calibrated by scanning a freshly cleaved highly ordered pyrolytic graphite (HOPG, SPI supplies, grade SPI-3, work function in air ∼4.6 eV), commonly used as a calibration method for KPFM.^51^ The KPFM images were taken with a scan rate of 0.9 Hz and processed with Park Systems XEI 1.8.5 image analysis software.

## 3 Results and Discussion

In this work, we propose the use of cationic lignin derivatives as new cationic conditioning agents to be incorporated into future hair care formulations. To assess their potential for hair conditioning, two cationic lignin derivatives with different physicochemical characteristics (Table 1) were prepared and their ability to interact with hair was then evaluated by studying their deposition onto model surfaces and human hair. Among the different models available that can mimic the characteristics of hair, we have selected SiO_2_ surfaces, which were subjected to oxidative pretreatment before use. For benchmarking purposes, a commercial polymer widely used in hair-care formulations, PQ11, was also characterized under identical conditions as those used for the lignin derivatives and employed as a petrochemical reference. The general structures of the monomers of the different polymers used in this work are represented in Figure 1.

**Figure 1:**
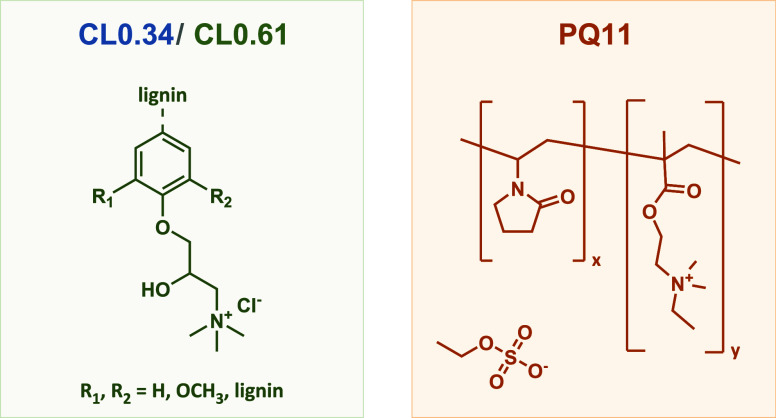
Chemical structures of cationic lignin (CL0.34/CL0.61) and PQ11. CL0.34 and CL0.61 differ from each other by the number of cationic substituent groups (i.e., 0.34 or 0.61) per aromatic moiety.

### 3.1 Studies of Polymer-Hair Interaction Using Model Surfaces

#### 3.1.1 Atomic Force Microscopy (AFM)

AFM is often used for morphological and topographical characterization of a surface at the nanoscale, but it is also a useful tool for assessing the adhesion properties of molecules on a surface. This technique can deliver useful insights regarding the homogeneity or heterogeneity of, for instance, an adsorbed polymeric layer through an easy and straightforward visual inspection of the resulting three-dimensional images, in addition to providing accurate information on the extension of the adsorption process. In this work, AFM was used to examine the topographic profile of the polymeric layers formed on top of the silicon wafers after deposition of CL0.34, CL0.61, and PQ11 solutions. The topographic images for each of the polymer solutions studied are presented in Figure 2. The corresponding height profiles can be found in Figure S1 (Supporting Information).

**Figure 2:**
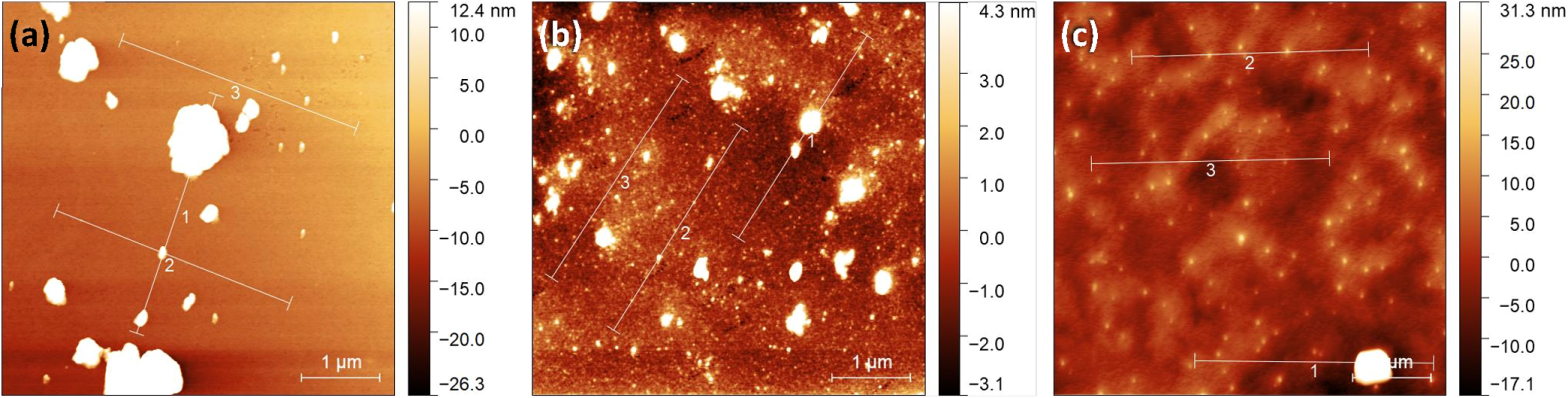
Topographic images of (5 × 5) μm^2^ sections of SiO_2_ surfaces coated with (a) CL0.34, (b) CL0.61, and (c) PQ11. AFM scans were obtained in tapping mode. The white lines indicate cross sections used to extract height profiles of the surface features; the corresponding profiles are shown in Figure S1 (Supporting Information).

The AFM images in Figure 2 reveal clear differences in the deposition of the various polymers on the wafers, suggesting possible different performances for the interaction and coating of hair. A highly heterogeneous coating was observed for CL0.34 (Figure 2a). For this polymer, large aggregates of several nm in length and height can be observed, randomly distributed across the surface. This suggests a high content of bulky insoluble material in the polymer solution, possibly combined with a strong tendency to aggregate during the drying process due to inherent low affinity toward the surface. The root-mean-square (RMS) roughness measured for this sample is 28.0 nm. Surfaces treated with CL0.61 and PQ11 show more similar profiles, in contrast to that of the CL0.34. These samples present surfaces that are more homogeneously coated, and the presence of larger aggregates is scarce. Apart from these few scattered clusters, the height of the peaks for the homogeneous region is typically lower than 5–15 nm, as can be observed for the height profiles (profiles 1 to 3, Figure S1b, and profiles 2 and 3, Figure S1c). The estimated RMS roughness values were 2.3 and 6.6 nm for CL0.61 and PQ11, respectively, which are significantly lower than that observed for CL0.34, highlighting their better spreading capability upon deposition on the surface. In fact, the topographical profiles of CL0.61 and PQ11 (Figure S1b,c) resemble that of a typical conditioning polymer on a model surface, with the polymer assembled in a mixture of interconnected groups that correspond to the bright areas with heights on the order of 4 nm.^41^ The difference between the results observed for CL0.34 and CL0.61 is somehow expected given the structural and physicochemical differences among these two derivatives. CL0.34 has a lower degree of cationization (DS = 0.34 ± 0.01 vs 0.61 ± 0.03) and ζ-potential (12.8 ± 0.4 mV vs 18.8 ± 0.3 mV) than CL0.61, which results in a lower water solubility and higher susceptibility to aggregation and precipitation. We believe this is the reason why the morphology of the CL0.61-coated surface is less heterogeneous, without the significative presence of globular-like aggregates observed for CL0.34. The similarity of the coatings and the ζ-potentials of CL0.61 and PQ11 (17.2 ± 0.9 mV) further support the correlation between the resulting topography of the adsorbed material and the surface charge. This has already been reported for other polymers; for instance, in the case of polymer–surfactant complexes composed of poly­(diallyldimethylammonium chloride) (PDADMAC) and sodium methyl cocoyl taurate (SMCT), it was observed that the deposition of the PDADMAC–SMCT complexes was more homogeneous for those aggregates showing a higher ζ-potential, and, consequently, a higher surface charge.^52^ This higher charge density of the aggregates results in a strong electrostatic repulsion between charged groups, which promotes better deposition on the surface leading to a higher coverage, while lower surface charge leads to a more disordered structure as a result of the sparse deposition of collapse aggregates.^52^ Similar trend was also found for the adsorption of PQ10 and polymer–surfactant complexes of PQ10 with the anionic sodium dodecyl sulfate (SDS) surfactant.^53^ PQ10 shows higher ζ-potential than the PQ10–SDS complexes, which resulted in a more homogeneous deposition onto SiO_2_ surfaces, with lower RSM roughness values, resulting in a more uniform and smoother surface. Highly charged polymers require a lower amount to efficiently cover the negatively charged surface. It is important to note the fact that the AFM images present two types of structural patterns: the presence of particles of lower height dispersed in the surface and some large clusters with higher height. This suggests that different deposition mechanisms are involved, as previously described in the literature, including the direct electrostatic attraction of the positively charged species to the negatively charged substrate (lower height) and the gravitational sedimentation of bigger aggregates (higher height).^54^

#### 3.1.2 Quartz Crystal Microbalance with Dissipation Monitoring (QCM-D)

The adsorption of the different polymers onto SiO_2_ surface was followed by QCM-D. This technique allows to estimate the adsorbed amount and the apparent hydrated layer thickness of the formed film, the so-called acoustic thickness (h_ac_), as well as gather information regarding the mechanical behavior of the adsorbed material. For this, solutions of the different polymers were prepared, and the kinetics of the adsorption and washout processes were followed by QCM-D. Aqueous solutions with a concentration of 0.5 wt % were selected based on the previous report of the maximum acoustic thickness observed for chitosan deposition at this concentration.^10^ The results for all three polymers, i.e., CL0.34, CL0.61, and PQ11, are summarized in Figure 3. Figures 3b–d show the shift in frequency of the quartz sensor, Δ*f*, normalized by the number of overtone, *n*, for three measured overtones (*n* = 3, 5, and 7) as a function of time. Similarly, Figures 3e–g show the shift in dissipation, Δ*D*, as a function of time for the same overtones. The results obtained for the other overtones up to *n* = 13 follow the same trend. Three different regions are visible in all plots, which are numbered and have alternating shades for ease of interpretation: in step (1), an initial baseline corresponding to the frequency of the base crystal immersed in the solvent is observed; step (2), highlighted with colored background, corresponds to the moment when the solutions of the cationic polymers are introduced into the measurement chamber. At this point, there is a sharp decrease in the Δ*f*/*n*, which is accompanied by an increase in the dissipation factor. This results from the deposition of the polymer onto the negatively charged surface, since the mass increase lowers the resonance frequency. After the steady state of the adsorption is reached, corresponding to Δ*f*/*n* becoming constant (end of step 2), the measurement chamber is flushed with the same solvent used to prepare the solution under study, which in this case is water. This process is schematized in Figure 3a. It is worth noting that pure water was used for solution preparation to provide a simplified and controlled baseline for comparing the intrinsic adsorption behavior of the polymers on the negatively charged surface. In real hair-conditioning conditions, the ionic composition may vary depending on the water source and formulation ingredients. Since ionic strength can screen electrostatic interactions and alter layer hydration, swelling, and rinsing stability, the present results should be interpreted as a reference case for future studies performed under controlled ionic conditions.

**Figure 3:**
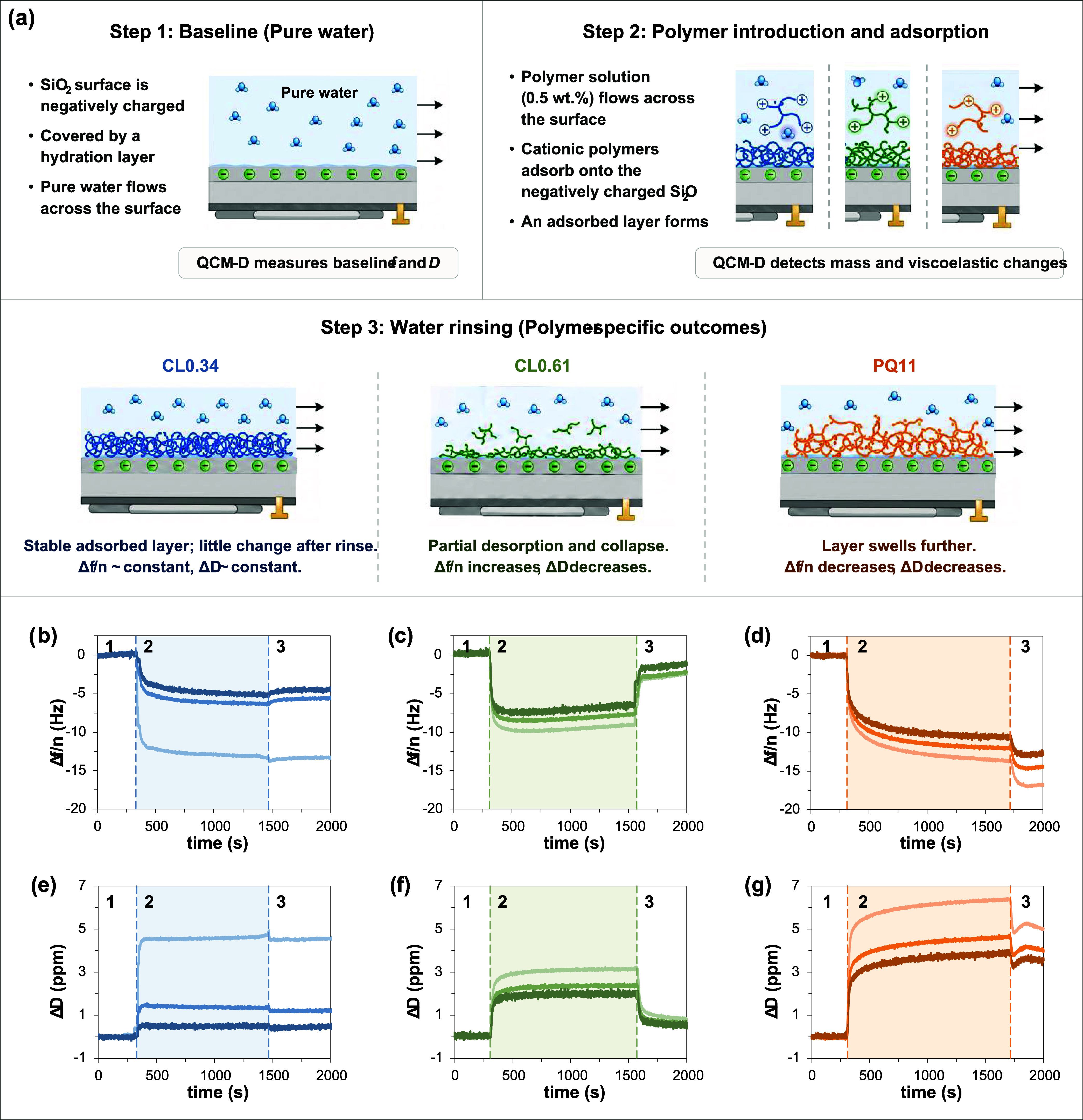
Adsorption kinetics and washout process for the adsorption of CL0.34 (left), CL0.61 (center), and PQ11 (right) onto SiO_2_ surface followed by QCM-D: (a) Schematic representation of QCM-D adsorption experiments on a negatively charged SiO_2_ surface. The SiO_2_ substrate contains surface negative charges covered by a hydration layer in water. Upon introduction of polymer solution, cationic polymers adsorb onto the hydrated surface. Blue (left panels), green (middle panels), and orange (right panels) chains represent CL0.34, CL0.61, and PQ11 polymers, respectively. Light blue clusters represent water molecules. Negative and positive symbols indicate surface and polymer charges, respectively. Figure 3a contains some AI-generated graphics created using AI-based text-to-image generation tools (OpenAI and Gemini). (b–d) shift of the central frequency, Δ*f*, normalized by the overtone number, *n;* (e–g) shift of dissipation, Δ*D*, of the different overtones, *n*. The color code is the same in all plots (light, medium, and dark curves correspond to the 3rd, 5th, and 7th overtones, respectively). The numbers and alternating colored regions within each plot indicate the different steps of the QCM-D experiments: (1) establishment of the equilibrium oscillation frequency of the quartz sensor immersed in water; (2) study of the adsorption of the polymer solution on the quartz sensor, and (3) rinsing the quartz sensor with the solvent (water).

In step (3), rinsing with water, performed at the same temperature as the measurement, mimics the rinsing process that occurs under real application in the shower and allows for the removal of weakly adsorbed material. Differences between the various polymers were observed during the rinsing phase, with the frequency remaining almost unchanged, increasing, or decreasing in the case of CL0.34, CL0.61, and PQ11, respectively. This suggests that the rinsing step affects the adsorbed layers differently. The increase in Δ*f/n* observed for CL0.61, in combination with the slight decrease in dissipation (Figure 3c and f), for example, can be explained by two concurrent phenomena. The first one is the desorption of chains that are weakly adsorbed to the surface, whereas the second one is related to the collapse of the layer upon rinsing. This effect is very subtle in the case of CL0.34 deposition, suggesting that the adsorbed layer is not as affected by rinsing as in the case of CL0.61. A different profile was observed for PQ11 (Figure 3d), where a further decrease in Δ*f*/*n* is visible. This suggests that rinsing may induce the swelling of the layer. It is worth noting that, during the injection and rinsing steps, fast shifts in Δ*f*/*n* were observed, which were not taken into account for the adsorption process analysis since they are considered experimental distortions from the design of the chamber.^9^

The results presented in Figure 3 can also give a qualitative understanding regarding the mechanical behavior of the adsorbed polymeric layers. The absence of overlap between the Δ*f/n* curves at different overtones suggests that the adsorption of all three cationic polymers onto negatively charged surfaces may lead to films with a viscoelastic character.^55,56^ This is further supported by the dissipation factor, Δ*D*, which increased during the adsorption process. In contrast to rigid films, which show little or no overtone dependence in frequency or dissipation, viscoelastic films may exhibit overtone-dependent responses.^57^ Shifts in dissipation reached values above 1 ppm (Figure 3e–g), which is commonly observed in films with substantial viscoelastic properties.^58^ This behavior is consistent across all polymer solutions, suggesting that lignin derivatives exhibit similar behavior to that of the commercial polymer.

A qualitative analysis of polymer deposition can be obtained from frequency shifts of the third overtone at the steady states reached after adsorption and subsequent rinsing with water. A comparison of these values is reported in Figure 4a. The results obtained for the two lignin derivatives are markedly different. While for CL0.61, a strong effect on the frequency shift upon rinsing is observed, with the frequency shift becoming less negative after rinsing, the change is negligible for CL0.34, with the frequency remaining almost unchanged. This may be explained by considering that the greater cationic charge of CL0.61 likely promotes its initial deposition on the negatively charged substrate, but the same structural feature may also enhance hydration and chain mobility in aqueous medium. As a result, rinsing in pure water can destabilize the outer, weakly associated fraction of the layer even if the initial electrostatic attraction was strong. This may be rationalized based on the different nature of the resulting layers, in agreement with the AFM results. CL0.34 forms a heterogeneous layer, which is compatible with a high fraction of trapped water within the adsorbed film due to a significant portion of the surface that remains uncovered by the polymer. Therefore, the rinsing process has little effect on the deposited layer. On the other hand, the higher homogeneity of the CL0.61 layer observed in AFM may be associated with structural contraction upon rinsing due to water expulsion, as supported by the strong reduction observed in the absolute value of frequency shift. It is worth noting that the higher absolute frequency shift observed for the CL0.34 compared to CL0.61, is consistent with its higher water content. As previously mentioned, PQ11 shows a different trend upon rinsing, with a shift toward more negative Δ*f/n* values. This suggests that the adsorbed PQ11 layer swells upon rinsing, leading to an increased apparent adsorbed mass. Qualitatively similar conclusions can be drawn from the analysis of the other overtones.

**Figure 4:**
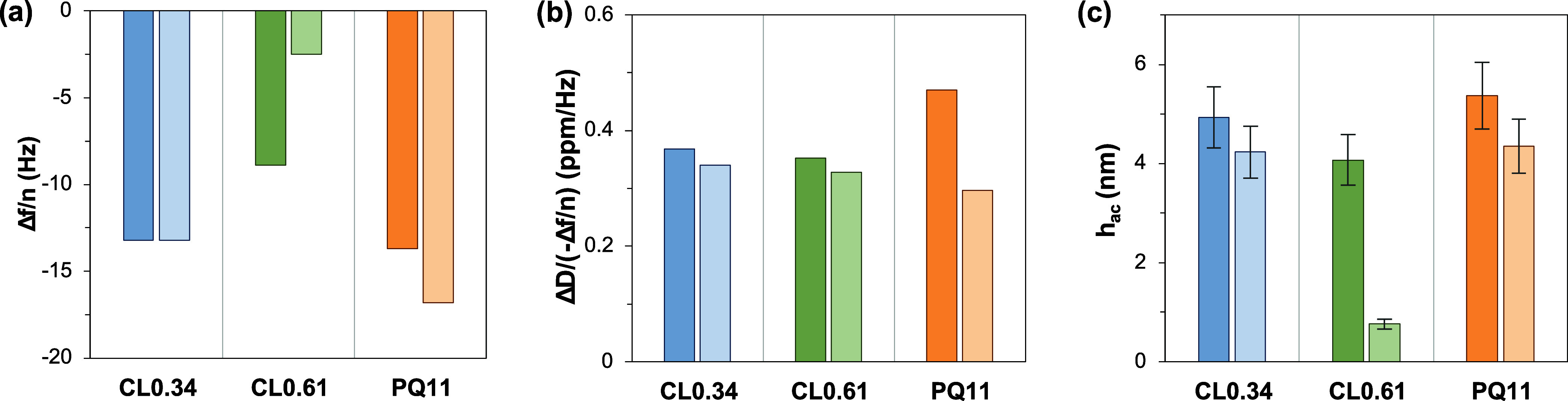
Results from the QCM-D experiments for CL0.34, CL0.61, and PQ11 adsorbed onto SiO_2_ surface, before (dark columns) and after (light columns) rinsing: steady-state values of (a) Δ*f*/*n* and (b) Δ*D/*(-Δ*f/n*), for the third overtone; (c) acoustic thickness of the adsorbed layers. The experiments correspond to the adsorption of a polymer solution with c ≈ 0.5 wt %.

The adsorption kinetics are important to understand how the polymer layer is formed. It involves an equilibrium between polymer–solvent and polymer–surface interaction forces. Conformational changes in the polymer also impact entropy and, therefore, affect the process dynamics.^59^ Qualitative insights on the energy/entropy balance of the adsorption process can be further obtained by analyzing the Δ*D*/(-Δ*f*/*n*) ratio. Higher values of this ratio indicate a stronger contribution of dissipation within the layer.^9^ This is related to the heterogeneity of the layer, which may arise either from polymer segments protruding into the aqueous phase or from the formation of heterogeneous layers dominated by isolated “islands” of polymer material across the surface.^9^ Figure 4b shows the steady-state values for the third overtone corresponding to the Δ*D*/(-Δ*f*/*n*) ratio upon layer adsorption and after rinsing. In this case, the ratio values are similar for all three polymers. Moreover, the results show a small decrease in the ratio upon rinsing, which is more pronounced for PQ11. Based on the Δ*D*/(-Δ*f*/*n*) ratio, it can be inferred that initially “fuzzy” layers are formed, which are subsequently compacted due to the expulsion of water upon rinsing.

From the frequency shift and dissipation data of the adsorption process shown in Figure 3, information about the thickness of the formed films can be extracted. Considering that there is no overlap among the different overtones, and that the dissipation shifts are relatively high, the quantitative analysis of the QCM-D data must account for the viscoelastic nature of the adsorbed layers. This requires the use of the Voigt-Voinova approach for data modeling.^60^ Using this model, the QCM-D data were analyzed to obtain an apparent hydrated layer thickness (h_ac_), which reflects the coupled polymer and solvent mass sensed by the crystal. Because the adsorbed films are viscoelastic and likely retain water, this thickness should not be interpreted as a dry geometric thickness of the polymer layer, but rather as a comparative measure of adsorption and hydration.^61^ Figure 4c shows the layer thickness obtained from the analysis of QCM-D experiments for films formed by the three cationic polymers (lignin derivatives and PQ11), both before and after rising with water.

As shown in Figure 4c, the film thickness values support the interpretation of the QCM-D response and provide an additional view of the layer build-up and stability upon rinsing. In fact, the layer thickness depends on the nature of the polymer. The results reveal that CL0.61 initially adsorbs onto the negatively charged surface, forming a relatively thick film, which can be related to its fuzzy character. However, upon rinsing with water, a strong decrease in the adsorbed amount is observed (Figure 4c). This is consistent with the frequency and dissipation shifts observed in Figures 3 and 4a, and suggest relatively weak adhesion of the lignin derivative to the negative surface. This behavior is not unexpected, as similar effects have been reported for other cationic polyelectrolytes adsorbed from solutions in pure water.^62^ Usually, the adsorbed amount can be increased by increasing the ionic strength of the medium.^10,62^ Although CL0.61 carries a higher charge density than CL0.34, its higher degree of cationization also increases its hydrophilicity and water solubility, which can favor stronger polymer–solvent interactions in low-ionic strength aqueous medium. Therefore, the higher initial adsorption observed for CL0.61 does not necessarily translate into stronger retention during rinsing. The rinsing step can remove weakly bound chains and promote contraction of the hydrated layer, which contribute to the observed decrease in the apparent adsorbed amount. Increasing ionic strength is therefore expected to enhance the deposition of CL0.61, as electrostatic screening promotes polymer–surface interactions over polymer–solvent interactions.^10^ In addition, at low ionic strength, charged polyelectrolytes typically adopt more flattened conformations upon adsorption, leading to thinner adsorbed layers.^63^

In the case of CL0.34, a higher layer thickness is observed, in good agreement with the AFM results; this can be ascribed to a high water content within the film. In addition, the lower charge density of CL0.34 compared to CL0.61, may lead to a more extended adsorption conformation of polymer chains, contributing to an increased layer thickness.^63^

The shear elasticity (μ) and viscosity (η) of the adsorbed films obtained from the analysis of the QCM-D data using the Voigt-Voinova model are shown in Table 3.

**Table 3:** Viscoelastic Parameters Obtained from the Analysis of the QCM-D Data for the Adsorbed Layers Following the Voigt-Voinova Approach

	before rinsing		after rinsing	
sample	μ (kPa)	η (mPa·s)	μ (kPa)	η (mPa·s)
CL0.34	57 ± 9	1.3 ± 0.3	62 ± 10	1.3 ± 0.4
CL0.61	75 ± 10	1.5 ± 0.4	72 ± 12	1.2 ± 0.4
PQ11	150 ± 18	1.3 ± 0.3	239 ± 15	1.4 ± 0.3

Overall, CL0.61 exhibited more favorable adsorption characteristics than CL0.34 on the negatively charged silicon surface. This conclusion is supported by its higher degree of cationization and ζ-potential, which were associated with a more homogeneous surface coverage and much lower roughness in AFM, as well as a clear and reproducible adsorption response in QCM-D. In contrast, CL0.34 formed a more heterogeneous layer with larger aggregates, higher roughness, and poorer spreading on the surface. Although CL0.61 showed some desorption/contraction upon rinsing, its initial deposition behavior and surface homogeneity suggest a better affinity for the substrate than CL0.34. It is worth noting that the adsorption of positively charged lignin derivates on the negatively charged silicon surface can be considered, in most cases, similar to that of other polycationic polymers in water, with the formed layer typically exhibiting a thickness below 5 nm.^64^ To enhance the deposition, it may be recommendable to increase the ionic strength (typical shampoo formulations contain NaCl in a concentration range 40–100 mM) or to include surfactants, which may promote synergistic effects in the adsorption.

### 3.2 Studies of Polymer-Hair Interactions Using Real Hair Samples

Aqueous solutions of CL0.61 and PQ11 were tested on real hair samples to further evaluate their conditioning performance. Bleached hair samples, either untreated or treated and subsequently rinsed, were visually examined to assess changes in appearance. As shown in Figure 5, the beneficial effect of applying a cationic solution to damaged bleached hair is clearly visible to the naked eye. Untreated bleached hair showed a frizzy appearance with poor definition, whereas both treated samples show improved alignment of the fibers and a striking reduction in frizz. Although no quantitative comparison between the two polymers can be made using this method, this preliminary qualitative assessment demonstrates the ability of both polymers to interact with the hair fibers and to provide a noticeable conditioning effect, even after rinsing.

**Figure 5:**
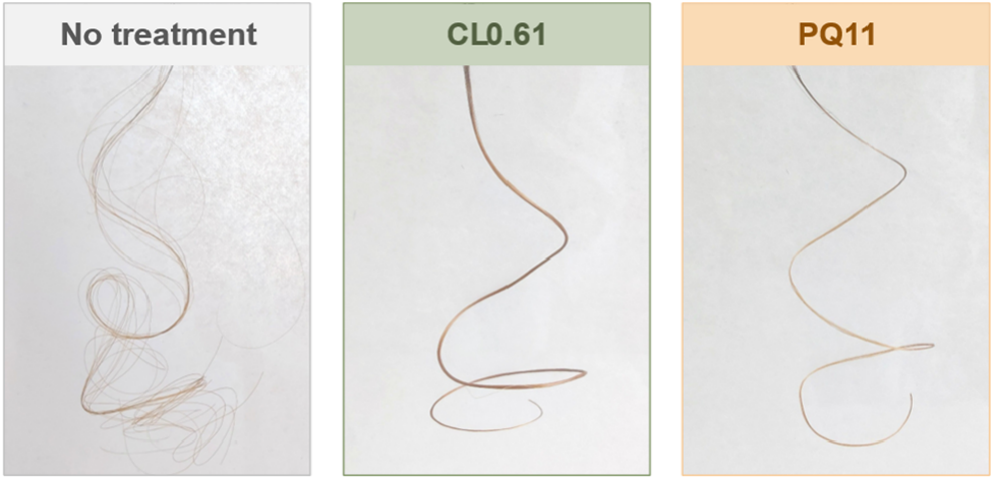
Visual appearance of bleached hair samples: untreated hair (left) and hair treated with aqueous solutions of CL0.61 (center) and PQ11 (right). Hair conditioning was performed by applying a 1 wt % polymer solution for 10 min followed by submersion in water for 2 min.

The structure and the surface potential of the hair samples were further investigated and mapped using the KPFM mode of an AFM apparatus. This analysis was performed to assess the effectiveness of hair treatment and the rinsing effect on damaged hair, by monitoring the shift of the surface potential from negative to positive values. The AFM-based Kelvin probe method has been used to simultaneously image the surface morphology and potential of human hair, and to track the charge distribution of ionized functional groups in biomolecular systems.^65–67^ Figure 6 shows the KPFM surface potential data and representative images of hair samples, including damaged hair, those treated with CL0.61 and PQ11, and their corresponding postrinsing states. As expected, the KPFM surface potential of the damaged hair was negative (i.e., median value of −17.6 mV). In contrast, the surface potential of hair samples treated with the cationic lignin derivative (CL0.61) or with the commercial PQ11 was reversed to positive, exhibiting potentials of about +175 and +18.8 mV, respectively. Compared with the commercial conditioning polymer (i.e., PQ11), the observed shift in surface charge density confirms the effectiveness of the developed lignin-based system as a hair conditioning agent. The corresponding quantitative surface potential maps for CL0.61 and PQ11 treated samples are shown in Figure 6c,e, respectively. As discussed above, and to mimic real-life conditions, the treated samples were further rinsed with water. After rinsing, the surface potential increased to +288 mV (CL0.61) and +271 mV (PQ11), confirming the persistence of cationic adsorbed species. However, the quantitative KPFM maps of rinsed samples (Figure 6d,f), reveal that the developed lignin-based conditioner induces a more uniform charge reversal across the hair surface compared to the commercial conditioner PQ11. Nevertheless, the validation of this hypothesis and its underlying mechanism requires further investigation. The corresponding surface morphology and roughness parameters are provided in the Supporting Information (Figure S2). The AFM analyses and the calculated roughness (Rq) indicate that the treatments lead to an approximately 2-fold increase in roughness. Rinsing the treated samples does not significantly affect roughness, despite the significant changes observed in the surface potential. These changes in roughness are likely related to intrinsic variability in the topography of individual hair fibers rather than to the conditioning process itself.

**Figure 6:**
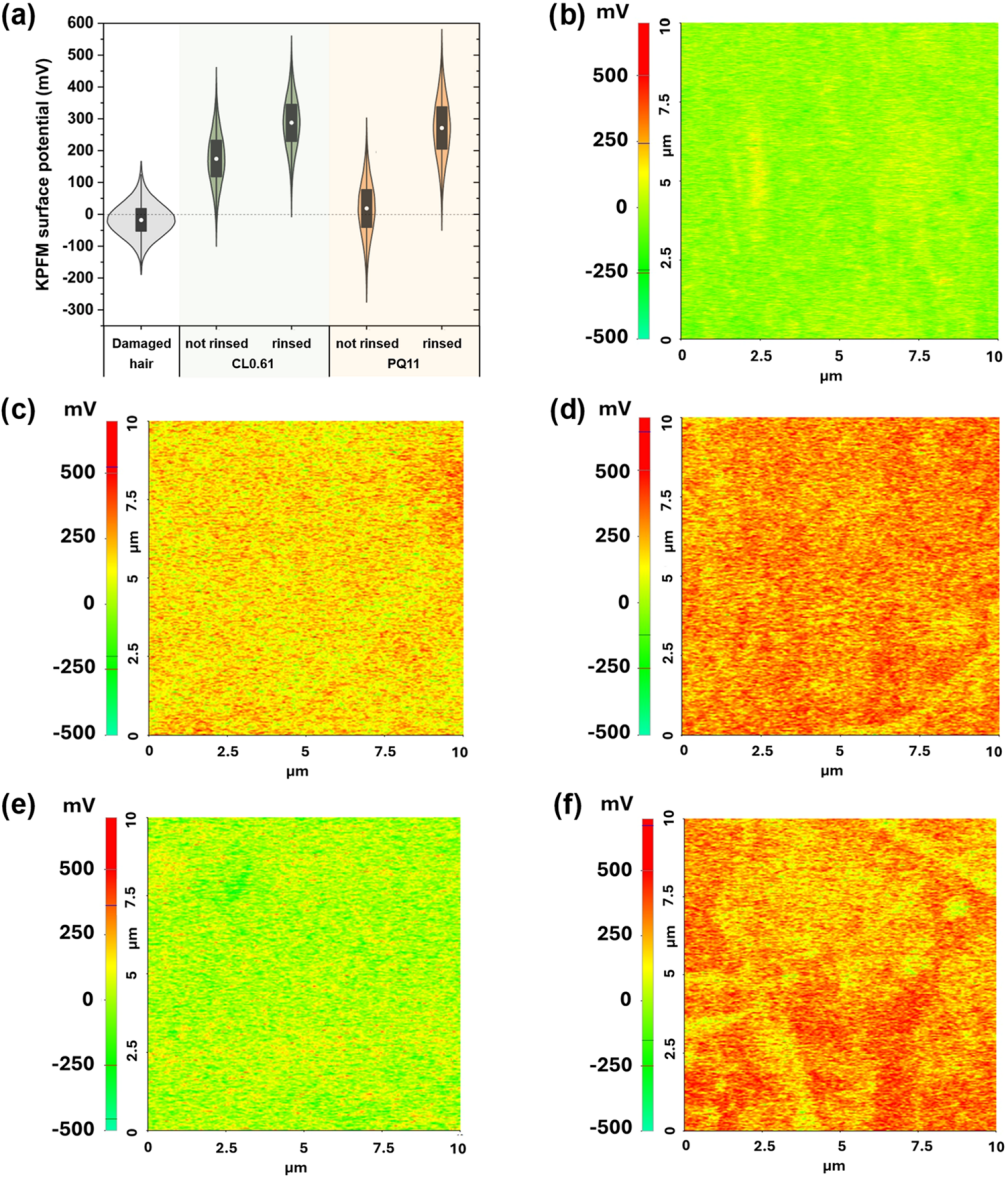
KPFM surface potential data and images of hair samples. (a) Violin with boxplots of the data (∼65,000 data points) for treated hairs, and (∼262,000 data points) for the untreated hair. The box represents the median (o) and 25/75 percentiles, and the whiskers represent the 5/95 percentiles. The KPFM surface potential images are shown for: (b) untreated hair, (c) not rinsed CL0.61-treated hair, (d) rinsed CL0.61-treated hair, (e) not rinsed PQ11-treated hair, (f) rinsed PQ11-tretead hair.

## 4 Conclusions

This work reports the study of cationic lignin polymers as potential new hair conditioning agents. Two cationic lignin derivatives (CL0.34 and CL0.61), prepared by CHPTAC etherification and exhibiting different degrees of cationization, were tested and compared with a commercially available polyquaternium ingredient, PQ11. Aqueous solutions of the three polymers were used to mimic a conditioning-and-rinsing process on model surfaces. The topography of coated silicon wafers was evaluated by AFM. QCM-D was used to understand the different deposition mechanisms of the tested polymers and to estimate the thickness of adsorbed layers. CL0.34, the less cationic lignin derivative, resulted in poor and highly heterogeneous surface coverage, showing large and randomly distributed aggregates across the surface. The presence of bulky insoluble aggregates suggests a low affinity of CL0.34 for the surface, indicating that low-charge lignin derivatives are not suitable for this application. In contrast, CL0.61 and PQ11 showed better spreadability and more homogeneous coatings of the silicon wafers, exhibiting topographical profiles typical of conditioning polymers. Results from QCM-D revealed that the adsorbed layers exhibit viscoelastic character and also suggest different behaviors between the polymers, particularly during the rinsing stage. The thickness of the adsorbed layers prior to rinsing was similar for the lignin derivatives and PQ11. CL0.61 was the most affected by the rinsing step, showing a noticeable decrease in adsorbed layer thickness. Two simultaneous processes are hypothesized to rationalize this behavior: desorption of weakly bound chains and the shrinking/contraction of the layer due to water expulsion. The low ionic strength of the medium (pure water) may promote desorption of highly soluble lignin chains. In the future work, it will be evaluated whether the deposition can be enhanced by increasing the ionic strength of the solution, or by incorporating the polymers into more complex formulations.

The application of CL0.61 and PQ11 solutions on real hair samples led to a visible reduction in frizz. Both polymers were effective in increasing the definition of bleached and frizzy hair. Untreated hair showed a negative median surface potential of −17.6 mV. After conditioning, a shift to positive values was observed, which further increased after rinsing. Similar surface potential values were achieved after conditioning with both polymers and subsequent rinsing: 288 mV for CL0.61 and 271 mV for PQ11.

This study demonstrates that CL0.61 exhibits adsorption behavior and performance comparable to that of the commercial cationic polymer PQ11. CL0.61 is capable of interacting with negatively charged surfaces, such as silicon wafers and damaged hair, and remaining adsorbed even after rinsing, as confirmed by the positive surface potential of treated hair. Its deposition and resulting layer thickness may, however, be improved by adjusting the ionic strength. This work combines a surface science proof-of-concept of the potential of cationic lignin to deposit on hair surface and it provides fundamental knowledge on the key polymer characteristics that govern the conditioning process. These insights are relevant for the future development of hair conditioner formulations, as they enable a preselection of the suitable polymers before any formulation work. Fundamental surface science approaches, such as the ones reported here, act as screening tools for a more cost- and time-effective formulation development. Future work will focus on the incorporation of suitable cationic lignin derivatives into prototype conditioner formulations with subsequent characterization and application on real hair samples. Given the abundance of lignin in nature and its wide availability as an industrial byproduct, combined with the currently well-established industrial adoption of the cationization of biopolymers, the scalability of this approach is a realistic expectation. Although further detailed studies are required, this work highlights the potential of novel cationic lignin derivatives for hair conditioning applications and contributes to widening the use of natural-based ingredients in the hair-care sector.

## Supplementary Material



## Notes

Supporting AFM data of model surfaces and hair samples coated with lignin derivates or the commercial cationic polymer (PDF)
