# Supplementary material for: Adsorption of Cationic Lignin Derivatives on Negatively Charged Model Surfaces and Hair Fibers: Implications for Hair Conditioning Performance
Source: ACS Appl Mater Interfaces. 2026 Jun 29;18(28):39510–22. doi: 10.1021/acsami.6c09520 (PMC13397484; doi:10.1021/acsami.6c09520)
Supplement: Supplementary file 1 [file am6c09520_si_001.pdf]

Supporting information

Adsorption of cationic lignin derivatives on negatively charged model surfaces and hair fibers: implications for hair conditioning performance

Catarina Fernandes[1,2*], Daniela Cabaça[1], Eduardo Guzmán[3,4], Ricardo Serra[5], Alireza Eivazi[6], Magnus Norgren[6], Luís Alves[1], Bruno Medronho[2,6], Maria da Graça Rasteiro[1], Carla Varela [1*]

[1] University of Coimbra, CERES, Department of Chemical Engineering, Pólo II – R. Silvio Lima, 3030-790 Coimbra, Portugal

[2] MED Mediterranean Institute for Agriculture, Environment and Development & CHANGE Global Change and Sustainability Institute, Universidade do Algarve, Faculdade de Ciências e Tecnologia, Campus de Gambelas, Ed. 8, 8005-139 Faro, Portugal

[3] Complutense University of Madrid, Department of Physico Chemistry-Plaza de las Ciencias s/n, 28040-Madrid, Spain

[4] Complutense University of Madrid, Pludisciplinar Institute-Paseo Juan XXIII 1, 28040-Madrid, Spain

[5] CEMMPRE Mechanical Engineering Department, University of Coimbra, 3030-788 Coimbra, Portugal

[6] Surface and Colloid Engineering, FSCN Research Center, Mid Sweden University, SE-851 70 Sundsvall, Sweden

*Corresponding author: csfernandes@uc.pt, carla.varela@uc.pt

1. AFM height profiles of model surfaces coated with the conditioning polymers

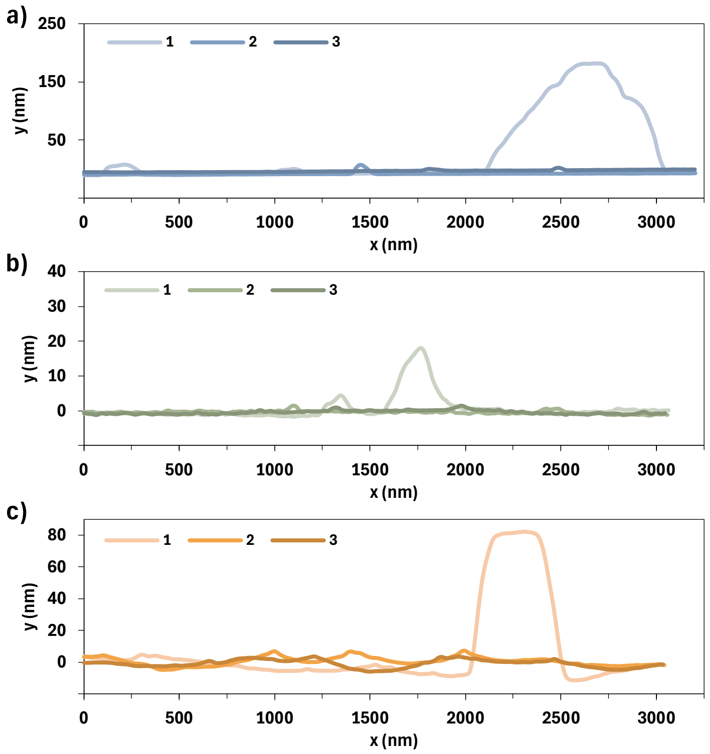

Figure S1. AFM height profiles extracted along the cross-sections indicated in Figure 1 for SiO_2 surfaces coated with **a)** CL0.34, **b)** CL0.61, and **c)** PQ11.

2. AFM data of hair samples treated with the conditioning polymers

The calculated surface roughness (Rq) of the untreated hair, not rinsed CL0.61-treated hair, rinsed CL0.61-treated hair, not rinsed PQ11-treated hair, and rinsed PQ11-treated hair were 33, 58.3, 54.4, 55.3, and 59.3 nm, respectively.

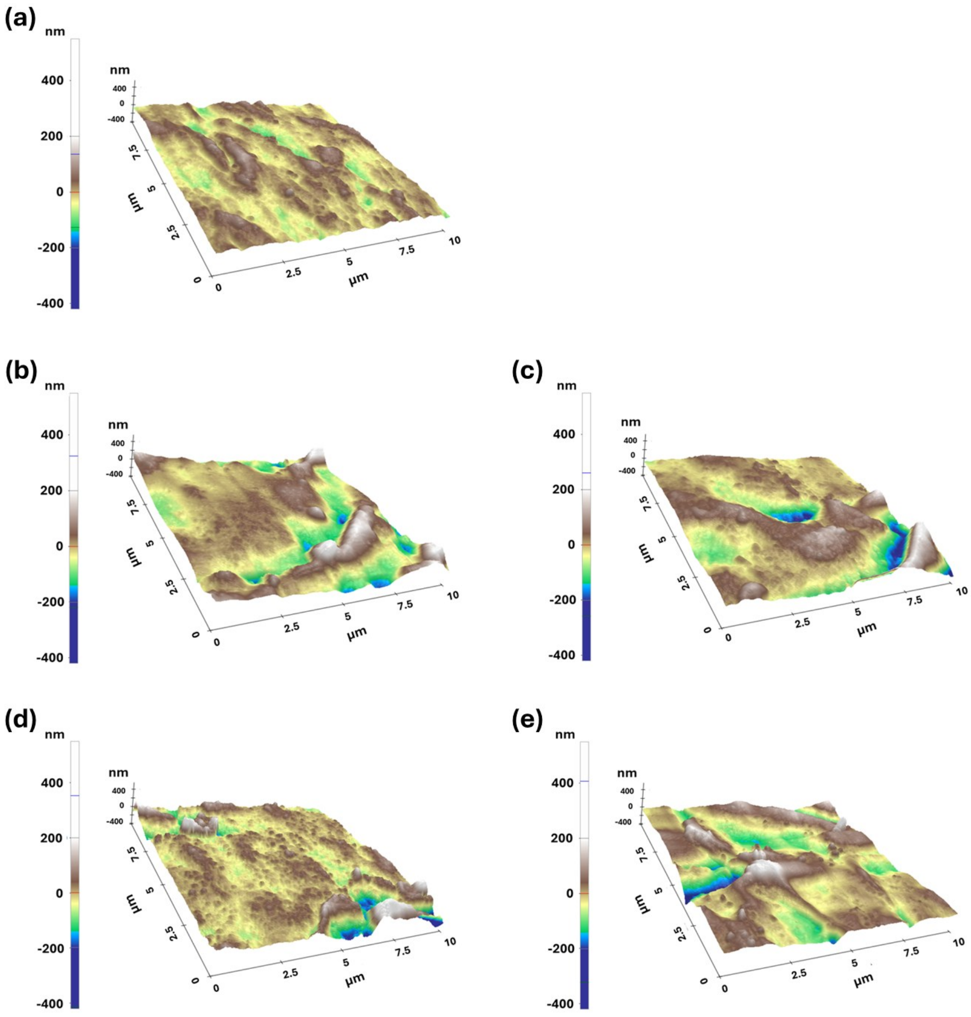

Figure S2. Atomic force microscopy (AFM) 3D height images of hair samples, (a) untreated hair, (b) not rinsed CL0.61-treated hair, (c) rinsed CL0.61-treated hair, (d) not rinsed PQ11-treated hair, and (e) rinsed PQ11-treated hair. Height scale bars are identical.
